# Families of microRNAs Expressed in Clusters Regulate Cell Signaling in Cervical Cancer

**DOI:** 10.3390/ijms160612773

**Published:** 2015-06-05

**Authors:** Luis Steven Servín-González, Angelica Judith Granados-López, Jesús Adrián López

**Affiliations:** 1 Laboratorio de microRNAs, Unidad Académica de Ciencias Biológicas, Universidad Autónoma de Zacatecas, Av. Preparatoria S/N, Zacatecas 98066, Mexico; E-Mails: stevenservin07@gmail.com (L.S.S.-G.); agranadosjudith@gmail.com (A.J.G.-L.); 2 Doctorado en Ciencias Básicas, Universidad Autónoma de Zacatecas, Av. Preparatoria S/N, Campus II, Zacatecas 98066, Mexico

**Keywords:** clusters, families of miRNAs, cell signaling pathways, cervical cancer

## Abstract

Tumor cells have developed advantages to acquire hallmarks of cancer like apoptosis resistance, increased proliferation, migration, and invasion through cell signaling pathway misregulation. The sequential activation of genes in a pathway is regulated by miRNAs. Loss or gain of miRNA expression could activate or repress a particular cell axis. It is well known that aberrant miRNA expression is well recognized as an important step in the development of cancer. Individual miRNA expression is reported without considering that miRNAs are grouped in clusters and may have similar functions, such as the case of clusters with anti-oncomiRs (23b~27b~24-1, miR-29a~29b-1, miR-29b-2~29c, miR-99a~125b-2, miR-99b~125a, miR-100~125b-1, miR-199a-2~214, and miR-302s) or oncomiRs activity (miR-1-1~133a-2, miR-1-2~133a-1, miR-133b~206, miR-17~92, miR-106a~363, miR183~96~182, miR-181a-1~181b-1, and miR-181a-2~181b-2), which regulated mitogen-activated protein kinases (MAPK), phosphatidylinositol-4,5-bisphosphate 3-kinase (PI3K), NOTCH, proteasome-culling rings, and apoptosis cell signaling. In this work we point out the pathways regulated by families of miRNAs grouped in 20 clusters involved in cervical cancer. Reviewing how miRNA families expressed in cluster-regulated cell path signaling will increase the knowledge of cervical cancer progression, providing important information for therapeutic, diagnostic, and prognostic methodology design.

## 1. Introduction

Cellular paths are controlled through a variety of mechanisms, since extracellular and intracellular signals are carried out by proteins, nucleotides, carbohydrates, ions like Ca^2+^, *etc.* A novel mechanism of cell path regulation is yielded by microRNAs, small RNA molecules that actively inhibit expression of all kind of proteins. Each miRNA is able to regulate a great number of proteins, which means these little molecules have great impact on cellular fate through cell pathway regulation. Reviewing how miRNA families are expressed in clusters and how they regulated cell path signaling will increase the knowledge of cervical cancer progression, giving advantages for therapeutic, diagnostic, and prognostic methodology design. Several works are enriching this knowledge by the generation of experimental evidence of miRNAs’ participation in regulation of cell paths. Therefore, coupling the information available we propose cell paths affected through miRNAs in cervical cancer. The advantages generated by gain or loss of function in miRNA expression on cancer development [1,2] could be due to the effect of one or more members of the family or cluster. This work shows several misregulated miRNAs that belong to miRNA families and clusters (Table 1), suggesting a complex system of regulation affecting genes and cellular processes.

**Table 1:** Families of miRNAs, grouped in clusters. The function of the cluster miR-17~92 is oncogenic except for miR-17. Function is defined as A for anti-oncomiR, O for oncomiR, and ? for unknown.

Clusters	miRNAs in the Cluster	Family of miRNAs	Chromosome	Function
miR-133a-2~1-1	miR-1-1 and miR-133a-2	miR-1 (1-1, 1-2 and 206); miR-133 (133a and 133b)	20	A/O
miR-1-2~133a-1	miR-1-2 and miR-133a-1		18	A/O
miR-133b~206	miR-133b and miR-206		6	O/?
miR-17~92	miR-17^−^, miR-18a, miR19a, miR-20a, miR-19b-1, miR-92-1	miR-17 (17, 18a, 18b, 20a, 20b, 93, 106a and 106b); miR-19 (19a, 19b-1 and 19b-2); miR-363 (363); miR-25 (25, 92a-1, 92a-2 and 92b)	13	O
miR-106a~363	miR-106a, miR-18b, miR-20b, miR-19b-2, miR-92-2 and miR-363		X	O
miR-106b~25	miR-106b, miR-93 and miR-25		7	O
miR-23a~27a~24-2	miR-23a, miR-27a and miR-24-2	miR-23 (23a, 23b and 23c); miR-24 (24-1 and 24-2); miR-27 (27a and 27b)	19	O
miR-23b~27b~24-1	miR-23b, miR-27b and miR-24-1		9	A
miR-29a~29b-1	miR-29a and miR-29b-1	miR-29 (29a, 29b-1, 29b-2 and 29c)	7	A
miR-29b-2~29c	miR-29b-2 and miR-29c		1	A
miR-34b~34c	miR-34b and miR-34c	miR-34 (34a, 34b and 34c)	11	A
miR-183~96~182	miR-183, miR-96 and miR-182	miR-183 (183); miR-96 (96); miR-182 (182)	7	O
miR-125a~let-7c~99b	miR-125a, let-7e and miR-99b	Let-7 (a-1, a-2, a-3, b, c, d, e, f-1, f-2, g and i); miR-99 (99a, 99b and 100); miR-125 (125a, 125b-1 and 125b-2)	19	O/?/A
miR-let-7c~99a	let-7c and miR-99a		21	?/A
miR-100~let-7a-2	miR-100 and let-7a-2		11	A/?
miR-181a-1~181b-1	miR-181a-1 and miR-181b-1	miR-181 (181a-1, 181a-2, 181b-1, 181b-2, 181c and 181d)	1	O
miR-181a-2~181b-2	miR-181a-2 and miR-181b-2		9	O
miR-181c~181d	miR-181c and miR-181d		19	O
miR-199a-2~214	miR-199a-2 and miR-214	miR-199 (199a-1, 199a-2 and 199b); miR-214 (214 and 3120)	1	A
miR-302s	miR-302a, miR-302b, miR-302c, miR-302d and miR-367	miR-302 (302a, 302b, 302c, 302d, 302e and 302f) miR-367 (367)	4	A

## 2. miRNAs Families Altered in Cervical Cancer

A gene family is a group of genes with a common phylogenetic origin and possible functional homology [3]. The members of miRNA families may be grouped in clusters expressed from a same transcript. However, it is important to note that not all miRNAs from a cluster are expressed at the same time even though the cluster is generated from a single transcript. Short clusters of miRNAs usually include two or three miRNAs and the larger are from four onwards [4]. The distance between miRNAs in a cluster could be from 1 to 10 Kb [5,6,7]. Additionally, most of the clusters have evolutionary conservation, implying an important biological function. It should be noted that paralogous miRNAs are located on different chromosomes; therefore, they should be expected to have differential regulation and expression [8]. Members of different miRNA families have evolved to target a diverse set of transcripts [9,10] regulating several cellular signaling pathways. It this work we point out the pathways regulated by families of miRNAs, grouped in 20 clusters, involved in cervical cancer.

## 3. Cell-Signaling Pathways Regulated by Members of miRNA Families Expressed in Clusters

### 3.1. Regulation of PI3K-AKT and MAPK Axis by miR-133b from the Cluster miR-206~133b

**Figure 1:**
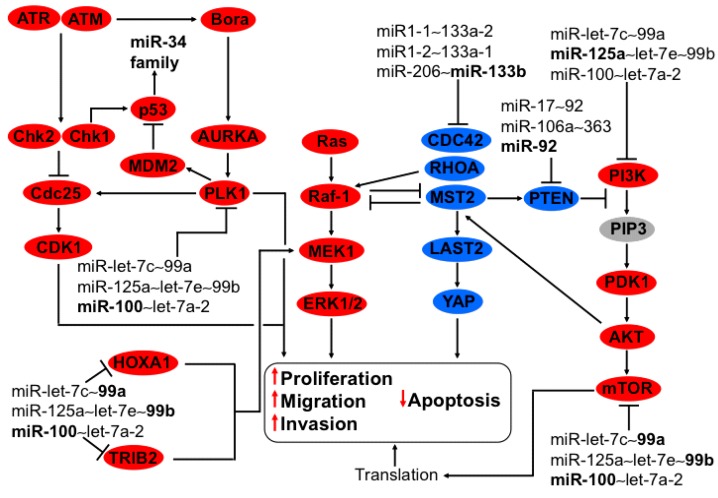
Regulation of MAPK, PI3K-AKT, and G2-M checkpoint by members of the family miR-125 and members of miR-let-7c~99a, miR-125a~let-7e~99b, miR-100~let-7a-2, and miR-206~133b clusters in cervical cancer. MAPK cell signaling is triggered by MEK-1 activation via TRIB2 and HOXA1 conducing to ERK1/2 phosphorylation, thereby provoking apoptosis reduction and inducing proliferation, migration, and invasion. This pathway is regulated by downregulation of TRIB2 and HOXA1 by the family miR-99 clustered in miR-let-7c~99a, miR-125a~let-7e~99b, and miR-100~let-7a-2. The members of these clusters are diminished in cervical cancer. MST2 inhibits Raf-1 and activates LAST2/YAP, reducing Ras-MEK-ERK activation, thus diminishing proliferation and inducing apoptosis, respectively. The oncomiR-133b from the cluster miR-133b~206 inhibits the tumor suppressor MST2. As well as CDC42 and RhoA that in turn inactivates Plexin B1 and PTEN increasing PI3K-PDK1-AKT-mTOR signaling, thus augmenting translation. MiR-125a from the cluster miR-125a~let-7e~99b counteracts the effect of miR-133b through the downregulation of PIK3CD, inhibiting PI3K-PDK1-AKT-mTOR signaling. The family miR-99 reduces mTOR protein expression, having an opposite effect to the miR-206~133b cluster. The cell cycle is arrested by DNA damage via ATM and ATR activation consecutively by the activation of Chk1/2 blocking CDC25 hampering CDK1 stopping transition from G2 to M. ATM and ATR prevent Bora activation conducing to Aurora A, PlK1 and MDM2 inactivation, thereby stabilizing p53 and activating miR-34 family transcription. Also, p53 is activated directly by Chk1/2, ATR, and ATM. In cervical cancer PlK1 is upregulated because miR-100 from the cluster miR-100~let-7a-2 is downregulated, promoting MDM2 increase and contributing to p53 decrease, with a concomitant miR-34 family shrink increasing protein translation, proliferation, and apoptosis reduction. In bold are the miRNAs with effect on genes with validated experimental data.

The cluster miR-133a-2~1-1 is formed by miR-1-1 and miR-133a-2 [11], localized on chromosome 20, having 10,536 nt of distance between them. In this sense miR-1-2 and miR-133a-1 could be expressed as a cluster since the distance between them is 3219 nt long and they are localized on chromosome 18. In a similar way, miR-133b and miR-206 are localized on chromosome 6 and the distance between them is 4607 nt long [6]. Interestingly, miR-1 and miR-133 from the clusters miR-133a-2~1-1 and miR1-2~133a-1 have opposite expression in cervical cancer: whereas miR-1 is downregulated, miR-133 is upregulated [12]. MiR-133b from the cluster miR-206~133b over-expression activates AKT1, ERK1, and ERK2 phosphorylation, inducing cell proliferation and colony formation in cervical cell lines by the degradation/inhibition of mRNAs and proteins of mammalian sterile 20-like kinase 2 (MST2), cell division control protein 42 homolog (CDC42), and Ras homolog gene family member A (RhoA) [13] (Figure 1). Proliferation and apoptosis are regulated through Raf-1 and MST2 activation. Raf-1 is activated by Ras inducing MEK1, ERK1, and ERK2 phosphorylation [14], while MST2 triggers apoptosis through PUMA and caspases activation. MST2–Raf-1 interaction inhibits MST2 activation while MST2-RASSF1A interaction activates MST2. RASSF1A and Raf-1 compete for binding sites within MST2 [14,15]. Apoptosis stimulus induces MST2-RASSF1A interaction, activating MST2. AKT phosphorylates MST2, inducing Raf-1 interaction and thereby releasing RASSF1A from the complex inducing MST2 inhibition [15]. Furthermore, RhoA and CDC42 inhibit ERK1/2 activation as well as AKT signaling through PTEN activation [16,17,18]; however, it should be noted that RhoA could activate the cell signaling PI3K/AKT and ERK1/2 by the activation of Plexin-B1 [19,20], and probably by CDC42 too (Figure 1). It seems that a cell-specific pathway axis activation or inactivation response is dependent on intensity and time [18]. The AKT and MAPK cell signaling pathways have an intricate and complex regulation to induce cancer that in part could be explained by miRNA family members’ misregulation. MiR-133a and miR-133b differ at a single 3′ terminal nt and have similar expression levels in cervical cancer [12]; therefore, it is possible that there is a similar type of regulation on AKT and MAPK signaling (Figure 1).

### 3.2. Regulation of CUL5, NOTCH, TNKS2, PTEN-PI3K-AKT Axis by miR-17~92 and miR-106a~363 Clusters

The families miR-17, miR-19, and miR-25 are expressed in three well-characterized clusters that have different localization on the human genome. The cluster miR17~92, localized on chromosome 13, contains miR-17, miR-18a, miR-19a, miR-20a, miR-19b-1, and miR-92-1. The cluster miR106a~363 is found on chromosome X and contains the miRNAs, 106a, 18b, 20b, 19b-2, 92-2, and 363. Cluster miR106b~25 is localized on chromosome 7, formed of miR-106b, miR-93, and miR-25 [6,21]. The miRNAs of these clusters are upregulated since early steps of cervical progression, as shown in the model previously proposed in a review by our group [12]—Except for miR-17-5p, which is downregulated, and miR-18, about which there is not sufficient information in cervical cancer.

**Figure 2:**
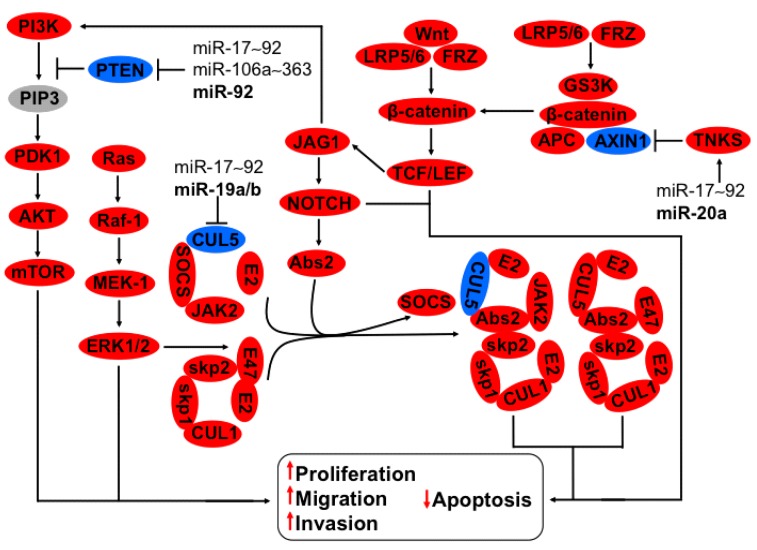
Regulation of CUL5, JAG-NOTCH, TNKS2, and PTEN-PI3K-AKT cell signaling by members of miR-17~92 and miR-106a~363 clusters in cervical cancer. E3 ubiquitin ligase complexes formed by CUL5-E2-SOCS-JAK2/3 and CUL1-skp1-skip2-E47-E2 are activated by Abs2 and SOCS interchange potentiated through JAG1-NOTCH axis. The cell signaling of GS3K-catenin-APC-AXIN-1-TCF/LEF is activated by AXIN-1 inhibition, which triggers the JAG1-NOTCH pathway, which in turn accelerates the interchange of SOCS by Abs2. Additionally, CUL1-skp1-skip2-E47-E2 is activated by phosphorylation of E47 via Ras-Raf-MEK-1-ERK1/2. The miR-19a and miR-19b grouped in the clusters miR17~92 and miR106a~363 downregulated CUL5, increasing proliferation, migration, and invasion and diminishing apoptosis. MiR-20a, a member of the cluster miR17~92, increases TNK2 expression that in turn inhibits AXIN-1, activating TCF/LEF, inducing JAG1-NOTCH1 signaling. Another member of the clusters miR17~92 and miR106a~363, miR-92, hinders PTEN expression, supporting PI3K-PDK1-AKT1 signaling thereby spreading proliferation, migration, invasion, and constraining apoptosis. In bold are the miRNAs with effect on genes with validated experimental data.

Xu *et al.* [22] showed that miR-19a/b from the cluster miR17~92 downregulates cullin 5 (CUL5) at the mRNA and protein level (Figure 2). CUL5 is a scaffold protein in E3 ubiquitin ligase complexes, which targets substrates for ubiquitin-dependent proteasome-mediated degradation [23]. The cullin-ring ligases form a bridge with the substrate-binding cytokine-inducible suppressors of cytokine signaling (SOCS) adaptor proteins and the E2 ubiquitin-conjugating enzyme [24]. Notch transcriptional signaling substitutes SOCS for ankyrin repeat and SOCS box-containing (Asb2) from the cullin-based complex constituted by S-phase kinase-associated protein 1 (skp1), skp2, CUL5, CUL1, E47, and E2 (Figure 2), inducing ubiquitination of its targets, which could be involved in differentiation and proliferation [25,26]. MAPK signaling is needed for E47 degradation [27] (Figure 2). A dual effect of these cell-signaling pathways is possible in cancer development: on one hand a turn-on of systems that favor tumor properties by phosphorylation, and on the other hand inducing ubiquitination-degradation of tumor suppressor genes by regulation of specific proteins of the ubiquitination-degradation system.

In line with this thought, miR-20a-5p, a member of the cluster miR17~92, upregulates tankynase 2 (TNKS2) in a sequence-dependent form inducing colony formation, migration, and invasion [28]. TNKS2 promotes wnt-catenin pathway liberating the inhibitory complex formed by -catenin, glycogen synthase kinase 3 (GSK-3), adenomatous polyposis coli (APC), and AXIN1 by AXIN1 degradation, inducing stabilization and freedom of -catenin activating transcription factor/lymphoid enhancer-binding factor 1 (TCF/LEF) [29]. Wnt-catenin activates Notch signaling through the JAG1 ligand of the Notch receptor [30], potentiating the hallmarks of cancer [31] (Figure 2).

The clusters miR17~92 and miR106a~363 are involved in the positive regulation of the PI3K-AKT signaling pathway. Mature miR-92, which could be formed from the cluster miR17~92 and/or miR106a~363, downregulates PTEN in cervical carcinoma cells, thus turning on PI3K-AKT signaling [32] (Figure 2). One of the most important points in the carcinogenesis process is the transduction of cell signaling, favoring the hallmarks of cancer. Interestingly, PI3K signaling is activated through Notch pathway a common oncogene in cervical cancer [33,34]. The members of the clusters miR17~92, miR106a~363, and miR106b~25 could potentially regulate CUL5, TNKS2, and PTEN because they have a seed sequence that hybridizes with their mRNA (microRNA.org, MIRDB, Target Scan). The clusters miR17~92, miR106a~363, and miR106b~25 function as onco-miRs, activating several cellular signaling pathways that induce cervical cancer development.

### 3.3. Regulation of uPA-PLG-MMP and JAG-NOTCH Axis by Members of miR-23b~27b~24-1 Cluster

**Figure 3:**
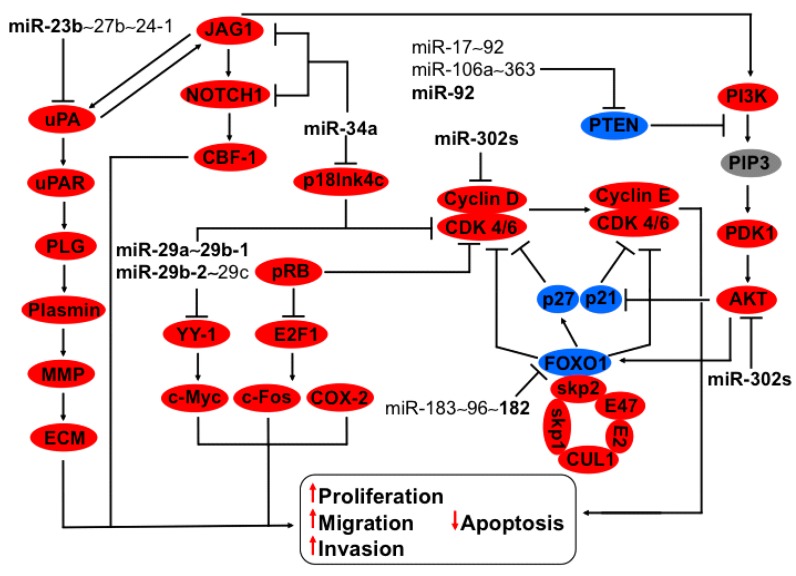
Regulation of JAG1-NOTCH1-uPA, Cyclin D-CDK4/6-p21-p27, and PI3K-PDK1-AKT1 cell signaling by members of the miR-23b~27b~24-1, miR-29a~29b-1, miR-29b-2~29c, miR17~92, miR183~96~182, and miR-302s clusters. MiR-34a, a member of the miR-34 family, downregulates JAG1 and NOCTH1 expression. JAG1 is activated by uPA inducing ECM reorganization via plasminogen-plasmin-MMP, inducing the hallmarks of cancer. Protein expression of uPA is decreased by miR-23b, a member of the cluster 23b~27b~24-1. The complex cyclin D-CDK4/6 phosphorylates RB inducing the liberation of E2F, favoring c-Myc, c-Fos and COX-2 transcription provoking cellular division. Cellular division progression is regulated by the inhibition of cyclin D via the miR-302s cluster and CDK4/6 through p18Ink4c, p21, and p27. The PI3K-PDK1-AKT1 axis decreases p21 and p27 protein expression, delivering CDK4/6 to be complexed with cyclin D. This signaling pathway is regulated by the downregulation of AKT due to the miR-302s cluster increasing p21 and p27 proteins, thereby inhibiting the complex cyclin D-CDK4/6. CDK4/6 is inhibited by p18Ink4c, which in turn is downregulated by miR-34a, inducing apoptosis and causing proliferation to diminish. Another point of control is given by the miR-29a~29b-1 cluster hindering CDK4/6, which provokes inhibition of the complex cyclin D-CDK4/6. YY-1, a target of the cluster miR-29a~29b-1, induces transcription of c-Myc in the absence of this cluster common in cervical cancer. Cell cycle continuity is dependent on the levels and the complex formed of cyclin D-CDK4/6 and cyclin E-CDK4/6. FOXO1 could directly hinder CDK4/6, thus impeding the formation and activation of the complex cyclin D-CDK4/6 and cyclin E-CDK4/6 and indirectly via p27. Cyclin D is indirectly upregulated by miR-182, one member of the cluster miR183~96~182, through FOXO1 downregulation conducing to proliferation, migration, invasion, and apoptosis induction. These cellular processes are enhanced via FOXO1 phosphorylation by PI3K-PDK1-AKT, which is recognized by skp2, a subunit of the skip1/cul1/F-box ubiquitin protein complex, targeting it to degradation via proteasomes. MiR-92 diminishes the expression of PTEN, triggering PI3K-PDK1-AKT signaling, which is conducive to FOXO1 reduction. In bold are the miRNAs with effect on genes with validated experimental data.

The families miR-23, miR-24, and miR-27 are organized in two clusters (Table 1). The cluster miR-23a~27a~24-2 is localized on chromosome 19 while cluster miR-23b~27b~24-1 is on chromosome 9 [4,6,35]. In cervical carcinoma cells it was shown that miR-23b diminished uPA expression via 3′-UTR mRNA binding [36] (Figure 3). This protein is a serine protease that degrades plasminogen to plasmin, thus activating metalloproteinases and inducing extracellular matrix (ECM) degradation. Recently it was shown that an increase of Notch receptors’ expression triggers the Notch pathway by uPA activation [37]. Additionally, JAG-Notch activation stimulates uPA transcription [38,39], inducing a mutual signaling activation exacerbated by miR-23b absence favoring cancer progression (Figure 3). The clusters miR-23a~27a~24-2 and 23b~27b~24-1 have a high similarity; however, it appears that the members of these clusters have opposite profile expressions: whereas miR-27a is upregulated, miR-23b and miR-27b are downregulated in cervical cancer [12]. MiR-23a, miR-27a, and miR-27b from the two clusters have sequences that may potentially regulate uPA (microRNA.org, MIRDB, and Target Scan). It remains to be addressed whether miRNAs of these clusters regulated the same targets to elucidate the participation of these clusters in cervical cancer’s advance.

### 3.4. Regulation of HSP47, YY-1 and CDK6 Axis by miR-29a~29b-1 and miR-29b-2~29c Clusters

The members of miR-29 family are divided into two clusters (Table 1). In the first one, miR-29a and miR-29b-1 are localized on chromosome 7, whereas in the second miR-29b-2 and miR-29c reside on chromosome 1 [6]. Interestingly the members of the family miR-29 have differential expression patterns in cervical cancer: while miR-29a and miR-29c are downregulated, the expression of miR-29b is not altered. The restoration of miR-29a inhibits mRNA and protein of Heat-shock protein 47 (HSP47) via 3′-UTR. This protein belongs to serpin, a superfamily of serine protease inhibitors and a molecular chaperone involved in the maturation of collagen molecules [40]. The overexpression of HSP47 could be crucial in the metastasis processes because structural and architectural changes are needed in the extracellular matrix for carcinogenesis. In a similar way, changes in the normal cell cycle are required in cancer. The proteins YY-1 and CDK6 are upregulated in CIN 1 and cervical cancer, respectively, by miR-29a and miR-29b, showing a fine-tuning regulation in the cervical carcinoma progress [12,41] (Figure 3). Given the similarity between miR-29a, b, and c, the members of the two clusters could regulate YY-1 and CDK6. It should be noted that the members of the miR-29 family have different effects on the cell cycle, indicating target specificity [42]. YY-1 regulates several genes related to cancer like HER2, COX-2, c-Myc, and c-Fox [43]. In addition, CDK-6, in complex with Cyclin D, phosphorylates and inhibits the interaction of pRB with E2F1, inducing target transcription and proliferation [44] (Figure 3).

### 3.5. Regulation of P18Ink4c and JAG-NOTCH-uPA Axis by miR-34a

P18Ink4c is a CDK4/6 inhibitor that is increased in CIN 2 and carcinoma but not in the normal cervix. *De novo* infection of human keratinocyte-derived raft tissue by oncogenic HPV increased p18Ink4c expression, suggesting it is one of the first oncogenes activated upon HPV infection. It is well known that oncogenic HPV reduces p53 expression as well as their effectors, like miR-34a, a member of the family miR-34. The validated targets of miR-34a participate in cervical cancer progression, as is the case with p18Ink4c [45], Jagged, and Notch1 affecting cell cycle and Notch signaling directly and indirectly through uPA, respectively [46] (Figure 3). miR-34 family members have a sequence similarity of more than 80%, so it is possible to have targets in common between miR-34a, miR-34b, and miR-34c, which are clustered on chromosome 11 and separated only by 418 nt [6]. Actually, Notch and Jag1 are potential targets of the cluster miR-34b~34c (microRNA.org, Target Scan, and MIRDB). The expression of this cluster could potentiate the downregulation of these proteins affecting Notch signaling, thus balancing proliferation/apoptosis signals.

### 3.6. FOXO1-Cullin-Rings Axis Are Regulated by the Cluster miR183~96~182

The cluster miR183~96~182 is localized on chromosome 7 [47]. MiR-182 and miR-183 are upregulated [48] while miR-96 is downregulated in cervical cancer [49]. The inhibition of miR-182 diminishes tumor growth and its overexpression inhibits apoptosis and increases the S phase cell cycle by downregulating the FOXO1 protein in cervical carcinoma [48]. FOXO1 is an inhibitor of cyclin/CDK complex like cyclin D1 and D2-CDK4 and cyclin E-CDK2 (Figure 3). FOXO1 phosphorylation by PI3K-AKT is recognized by skp2, a subunit of the skip1/cul1/F-box protein ubiquitin complex, targeting it to degradation via proteasomes [50] (Figure 3). The function and relation of miR-182 and FOXO1 seems primordial in carcinogenesis because this axis regulates several complexes that direct the cell cycle’s advance [51].

### 3.7. Regulation of PI3K-AKT and MAPK Axis by miR-let-7c~99a, miR-125a~let-7e~99b, and miR-100~let-7a-2 Clusters

miR-99 and miR-125 families are evolutionarily related and are clustered with members of the family let-7. Interestingly, miR-125a, let-7e, and miR-99b are localized on chromosome 19, separated only by 468 and 642 nt, respectively. Let-7c and miR-99a are clustered on chromosome 21, separated by 739 nt, as well as miR-100 and let-7a-2, which are grouped on chromosome 11 with 5707 nt of distance between them. MiR-99a and miR-125b-2 are localized on chromosome 21, separated by 52,068 nt; likewise miR-100 and miR-125b-1 are on chromosome 11 with 52,385 nt of distance between them. Whether these miRNAs are expressed as a cluster is not known; however, they regulate similar genes [6] impacting on common cell signaling.

MiR-99a inhibits protein synthesis of tribbles pseudokinase 2 (TRIB2), Homeobox A1 (HOXA1), and mechanistic target of rapamycin (serine/threonine kinase) (mTOR) via 3′-UTR [52] (Figure 1). Potentially TRIB2 could be also regulated by miR-125b-2 (MIRDB and Target Scan). The expression of miR-99a and miR-125b-2 is downregulated in cervical cancer [12], causing TRIB2, HOXA1, and mTOR overexpression probably by the combination of both miRNAs’ loss. The oncogene TRIB2 is increased in several carcinomas [53,54], probably controlling the specificity of the activation of MAPK [55] and increasing MAPK signaling (Figure 1). In a similar manner, the transcriptional factor HOXA1 triggers MEK1 mRNA and protein increase together with ERK1/2 phosphorylation [56], potentiating MAPK signaling and enhancing the cancer hallmarks. To exacerbate the capabilities of tumor cells, the mTOR pathway is constantly activated, generating translational protein increase (Figure 1). Additionally, miR-99b downregulates HOXA1 and mTOR [57]. In cervical cancer the expression of miR-99b and miR-125a are opposite [12], suggesting a specific selection of miR-125a over miR-99b, resulting in HOXA1 and mTOR overexpression. miRNAs’ binding capacity does not always result in protein synthesis inhibition, as is the case of miR-20a on TNSK2 regulation [28].

miR-100 and miR-125b have similar profile expression, pointing to an anti-oncogenic function. miR-100 of the cluster miR-100~let-7a-2 and miR-125b-1 are downregulated in the early steps of cervical cancer progression [12]. In this sense, miR-100 inhibits PLK1 [58], a protein that participates in G2/M phase check-point regulation to block cell progression induced by DNA damage; however, some tumor cells override this check-point [59]. Upon UV-DNA damage, the kinases ATM and ATR are activated, inducing Chk1/2 phosphorylation and activation and thereby triggering CDC25 phosphatase inhibition, resulting in CDK1 phosphorylation and G2/M arrest. Additionally, ATM/ATR inactivates Bora, leading to cascade inhibition of Aurora A, PLK1, and CDC25 (Figure 1). The overexpression or constant activity of PLK1 is conducive to MDM2 activation and concomitant p53 degradation via ubiquitination. Likewise, CDC25 activation and CDK1 dephosphorylation favor G2/M progression [60]. Interestingly, miR-100 regulates these cell-signaling pathways, making it a new player in the G2/M checkpoint. The profile expression of the cluster miR100~125b-1 and the similarity between them point to the fact that miR-125b might participate in this signaling, too. Other important targets of miR-100 are phosphatase (CTD (carboxy-terminal domain, RNA polymerase II, polypeptide A) small phosphatase-like) (CTDSPL), enzyme *N*-Myristoyltransferase 1 (NMT1), Transmembrane Protein 30A (TMEM30A), and chromatin remodeler SWI/SNF Related, Matrix Associated, Actin Dependent Regulator Of Chromatin, Subfamily A, Member 5 (SMARCA5), HOXA1, and mTOR. MiR-125b inhibits the PI3K/AKT pathway through downregulation of mRNA and protein PIK3CD via 3′-UTR binding, which is conducive to protein kinase A (AKT) and mTOR phosphorylation, inducing tumor growth volume inhibition [61] (Figure 1). Downregulation of the targets of the families and clusters of miR-99 and miR-125 hinders proliferation and migration, highlighting the importance of function loss in these families in cervical carcinogenesis progression. The imbalance of signaling pathways gives advantages toward development of cancer.

### 3.8. Regulation of PI3K-AKT and MAPK Axis by miR181a-1~181b-1 and miR181a-2~181b-2 Clusters

**Figure 4:**
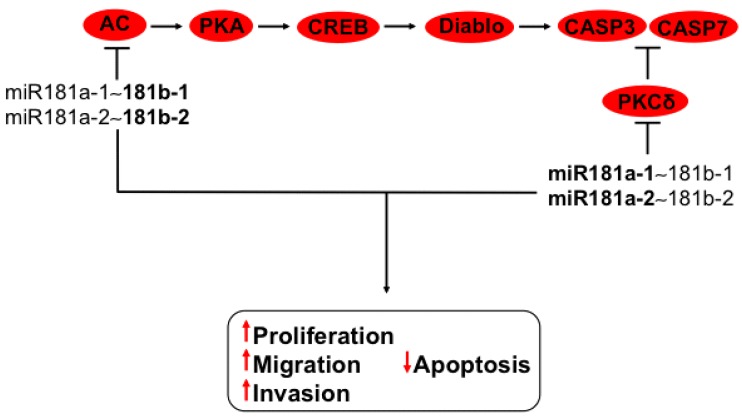
Regulation of AC-PKA-smac-caspase3 and PKC cell signaling by members of miR-181a-1~181b-1 and miR-181a-2~181b-2 clusters. Adenylyl cyclase (AC) induces PKA activation, generating CREB phosphorylation, which turns on smac/Diablo, causing caspase 3 activation. In addition, PKC activates caspase 3 and caspase 7. miR-181b, localized in clusters miR-181a-1~181b-1 and miR-181a-2~181b-2, downregulates AC; miR-181a also inhibits PKC protein expression. In bold are the miRNAs with effect on genes with validated experimental data.

The miR-181 family is clustered on chromosomes 1, 9, and 19. MiR-181a-1 and miR-181b-1 are localized on chromosome 1, while miR-181a-2 and miR-181b-2 are on chromosome 9 and miR-181c and miR-181d are located on chromosome 19 [6]. Cluster miR-181a~181b presents advantages for cervical cancer development. It has been reported recently that miR-181a confers radiochemo-resistance by diminishing mRNA and proteins of PKC via 3′-UTR binding, therefore decreasing caspase 3/7 activity and hindering apoptosis [62,63] (Figure 4). miR-181b downregulates adenylyl cyclase (AC), restricting cAMP production and promoting cell proliferation and apoptosis diminution [64]. cAMP production is conducive to PKA activation, which induces transcription of smac/Diablo by CREB, which leads to caspase activation [65] (Figure 4). This pathway seems to be regulated by all members of the miR-181 family clustered on distinct chromosomes—In agreement with their similarity [66] and with *in silico* predictions (microRNA.org, MIRDB, and Target Scan).

### 3.9. Regulation of MEK, JNK and Bcl2-l2 Axis by the miR-214~199a-2 Cluster

**Figure 5:**
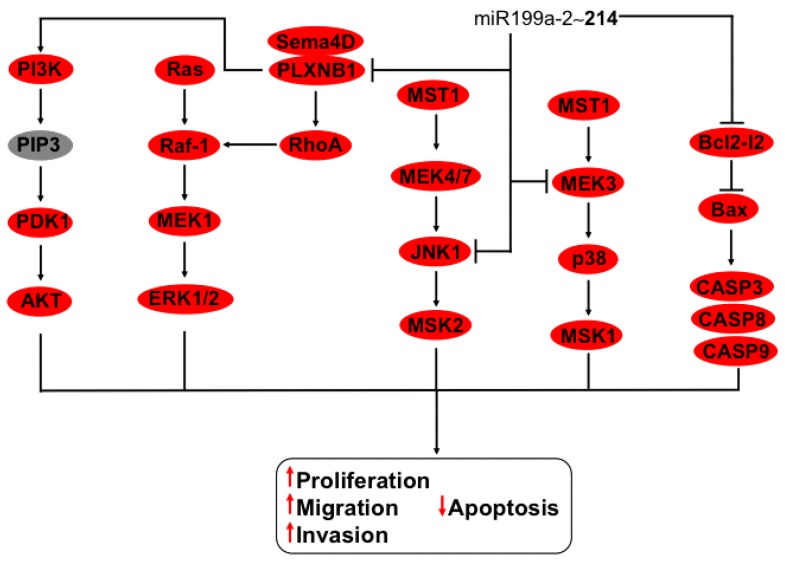
Regulation of Bcl-12l2-Bax-CASPS, MST1-MEK-4/7-JNK-1-MSK2, MST1-MEK-3-p38-MSK1, PI3K-PDK1-AKT, and Ras-Raf-MEK1-ERK1/2 cell signaling by the miR-214~199a-2 cluster. The anti-oncomiR-214 from the cluster miR-214~199a-2 promotes apoptosis by downregulation of Bcl-12-l2, permitting Bax activation with a consequent activation of caspases 9, 8, and 3. Furthermore, miR-214 inhibits JNK1 and MEK3, participants of cell signaling MST1-MEK-4/7-JNK-1-MSK2 and MST1-MEK-3-p38-MSK1, respectively. In addition, miR-214 controls MAPK activation by the downregulation of Plexin-B1, which in contact with Sema 4D activates RhoA, which then activates Raf-1 triggering MEK-1 and ERK1/2 phosphorylation together with PI3K-PIP3-PDK1-AKT, promoting the hallmarks of cancer. In bold are the miRNAs with effect on genes with validated experimental data.

Family miR-199 is formed of miR-199a-1, miR-199a-2, and miR-199b [6]. One member of family miR-199, miR-199a-2, is clustered with miR-214 [67,68] on chromosome 1, separated by 5628 nt [6] (Table 1). MiR-214 functions as anti-oncomiR in cervical cancer in this respect; it is not known if miR-199a-2 has the same role or if it participates in cellular pathways regulated by miR-214. The targets of miR-214 are implicated on several cellular pathways. For example, signaling through trans-membrane receptor Plexin-B1 induced cell survival, proliferation, angiogenesis, invasion, and metastasis in cervical cancer [69]. Semaphorin D4 binds to Plexin-B1, inducing the activation of RhoA, which in turn activates the Raf protein, triggering MEK/ERK signaling [20]. Cell-signaling pathway MEK/JNK is inhibited by miR-214 targeting MEK3 and JNK1, decreasing cell proliferation [70]. Importantly, these pathways have different targets. On one hand, MEK3 induces p38 activation by phosphorylation, resulting in MSK1 triggering. On the other hand, JNK1 activates MSK2 (Figure 5). Additionally, miR-214 reduces GALNT-7 protein expression, affecting proliferation, migration, and invasion in cervical cell lines [71], and controls cell death through mRNA and protein downregulation of anti-apoptotic proteins like Bcl-2l2, which in turn induces Bax increment and caspase 9, 8, and 3, the activation of which triggers intrinsic/extrinsic apoptosis pathways [72] (Figure 5).

### 3.10. Regulation of Cyclin D and AKT Axis by the miR-302s Cluster

The family miR-302 is formed of miR-302 a, b, c, d, e, and f. MiR-302a, miR-302b, miR-302c, and miR-302d are clustered on chromosome 4, while miR-302e and miR-302f are on chromosomes 11 and 18, respectively [6]. Cluster miR-302s, formed of miR-302a, miR-302b, miR-302c, miR-302d, and miR-367, downregulates AKT and Cyclin D at the protein level via 3′-UTR interaction (Figure 3). Notably, it was shown that AKT phosphorylation could be affected by miR-302 overexpression, independently of PTEN, whose expression was not affected by this miRNA cluster. Furthermore, protein levels and AKT phosphorylation diminution resulted in a p27 and p21 increase [73] (Figure 3).

## 4. Conclusions

In this work we identified 20 clusters implicated in cervical cancer; based on the literature reviewed, these were known to regulate several cell signaling pathways governing carcinogenesis. The cell pathways PI3K-AKT-mTOR, MST1-MEK-4/7-JNK-1-MSK2, MST1-MEK-3-p38-MSK1, and Ras-Raf-MEK-ERK are activated or inhibited by anti-oncomiRs clusters (let-7c~miR-99a, miR-125a~let-7e~99b, miR-100~let-7a-2, miR-199a-2~214, and miR-302s) and oncomiRs (miR-133a-2~1-1, miR-1-2~133a-1, miR-133b~206, miR-17~92, miR-106a~363, and miR183~96~182). The anti-oncomiRs and oncomiRs expressed in these clusters are downregulated and upregulated, respectively, since early steps of cervical cancer progression. However, it should be noted that miRNAs’ processing and stability are unique because we found exceptions like miR-17-5p and miR-1 that must be down- or upregulated upon cluster expression in cervical cancer (Table 1). The complex regulation of the anti-oncomiRs (miR-34a and cluster 23b~27b~24-1) and oncomiRs clusters (miR-17~92 and miR-106a~363) could be noted in the connection between the NOTCH axis and the proteasome system, which are connected and modulated by PI3K-AKT-mTOR and Ras-Raf-MEK-ERK. In a similar fashion, the cell cycle phases G1 and G2-M and apoptosis are balanced by anti-oncomiRs (miR-34a, miR-29a~29b-1, miR-29b-2~29c, miR-214, and miR-302s) and oncomiRs (miR-17~92, miR-106a~363, miR183~96~182, miR-181a-1~181b-1, and miR-181a-2~181b-2). The works we reviewed here report mature miRNAs. In this context we are missing data regarding the cluster origin and function of all the miRNAs expressed in the clusters. Do clusters on different chromosomes have equal transcript processing and stability levels? The provenance of miRNAs should be taken into account for therapy development. With this in mind, eight clusters function like oncomiRs and nine as anti-oncomiRs. Three clusters have dual activity as anti-oncomiRs and oncomiRs. This work highlights the clusters involved in cell signaling pathway regulation that could be used in therapy, diagnosis, and prognosis.
